# Progress in understanding and overcoming biomass recalcitrance: a BioEnergy Science Center (BESC) perspective

**DOI:** 10.1186/s13068-017-0971-1

**Published:** 2017-11-30

**Authors:** Paul Gilna, Lee R. Lynd, Debra Mohnen, Mark F. Davis, Brian H. Davison

**Affiliations:** 1 BioEnergy Science Center, Oak Ridge National Laboratory, Oak Ridge, TN 37831 USA; 2 Biosciences Division, Oak Ridge National Laboratory, 1 Bethel Valley Road, Bldg. 1505, Rm. 100A, Oak Ridge, TN 37831-6037 USA; 3 Thayer School of Engineering, Dartmouth College, Hanover, NH 03755 USA; 4 Complex Carbohydrate Research Center and Department of Biochemistry and Molecular Biology, University of Georgia, Athens, GA 30602 USA; 5 National Bioenergy Center, National Renewable Energy Laboratory, Golden, CO 80401 USA

**Keywords:** Bioconversion, Bioenergy, Recalcitrance, Center operation, Biomass

## Abstract

The DOE BioEnergy Science Center has operated as a virtual center with multiple partners for a decade targeting overcoming biomass recalcitrance. BESC has redefined biomass recalcitrance from an observable phenotype to a better understood and manipulatable fundamental and operational property. These manipulations are the result of deeper biological understanding and can be combined with other advanced biotechnology improvements in biomass conversion to improve bioenergy processes and markets. This article provides an overview of key accomplishments in overcoming recalcitrance via better plants, better microbes, and better tools and combinations. A perspective on the aspects of successful center operation is presented.

## Background

Biomass recalcitrance—the resistance of plants to release their sugars for fermentation or upgrading—is a primary barrier to efficient and economical production of advanced biofuels [1, 2]. Overcoming and understanding recalcitrance was the unifying vision of the US Department of Energy (DOE) BioEnergy Science Center (BESC), now in its final and 10th year of operation. The mission of BESC was “*to enable the emergence of a sustainable cellulosic biofuels industry by leading advances in science and science*-*based innovation resulting in removal of recalcitrance as an economic barrier to cost*-*effective production of biofuels* [3].” Due to advances in biotechnology, BESC believed that biological solutions were the most promising path by which to achieve these breakthroughs. In response to a DOE challenge [4], Oak Ridge National Laboratory (ORNL) led the formation of BESC by gathering experienced researchers from multiple US institutions, who had been separately interested in separate aspects of overcoming biomass recalcitrance targeting advanced biofuels and specifically cellulosic ethanol.

Recalcitrance began as an operationally defined phenotype. With both applied and fundamental goals, BESC perceived that we needed to transform the understanding of recalcitrance; this required detailed knowledge of the chemical, structural, and physical properties of biomass and how these properties influenced deconstruction by enzymes and thermophilic microorganisms. This search led to altering plant cell wall properties by manipulating key plant polymer biosynthetic pathways, which led to studies of the interactions of the plant cell walls and the enzymes and microbes during deconstruction and fermentation. The BESC team has redefined recalcitrance so that now recalcitrance is on the path to being an understandable and manipulatable set of properties based on cell wall formation and bioconversion. A key outcome of the BESC team’s approach was to transform understanding in both fundamental and operational impacts to strategies that will eliminate recalcitrance as an economic barrier to commercialization.

**Fig. 1:**
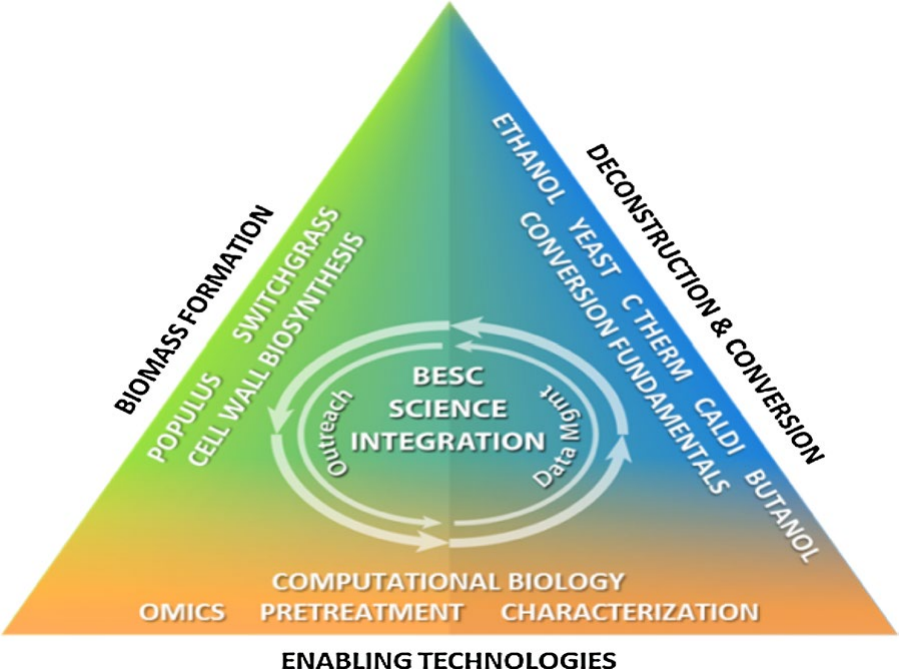
BESC was organized into three integrated areas with multiple targets

This singular focus on recalcitrance science was BESC’s hallmark worldwide. BESC was organized into three areas: Biomass Formation and Modification, Biomass Deconstruction and Conversion, and Enabling Technologies (Fig. 1). All three areas included both fundamental understanding and complementary proof-of-concept components. Our ability to design, conduct, and analyze wide-ranging campaigns, along with our effective communications and capacity to integrate cross-disciplinary teams within the BESC organization, has been key to our success in scientific areas that are critical to overcoming the formidable biological and technological barriers that biomass recalcitrance presents.

## Discussion: major accomplishments to date

From late 2007 to fall 2017, BESC published more than 945 journal articles, 10% in high-profile journals (impact factor > 9) and advanced the education of more than 230 professionals, who are now productive members of the bioeconomy workforce. More details with respect to the output of BESC and the other two USDOE Bioenergy Research Centers are in Slater et al. [5].

### Biomass formation

*Populus* and switchgrass (*Panicum virgatum*) were the chosen feedstocks for studies of cell wall-related genetic modifications that could impact recalcitrance and inform understanding. Both are high yield perennials recognized as potential domestic biofeedstocks [4, 6]. Populus was the first sequenced woody feedstock [7]. Switchgrass is a native herbaceous perennial that could grow on marginal land. Both were deemed tractable for studies aimed at determining the basis of, and ameliorating, recalcitrance. Key advances in biomass formation led by BESC include:

- Significant advances were made in understanding, manipulating, and managing plant cell wall recalcitrance and conversion. We showed that multiple plant genes control cell wall recalcitrance, and that manipulation of these genes can yield lower recalcitrance perennial biofeedstocks [8]. This included increasing our understanding of the cell wall structure and biosynthetic pathways for lignin, xylan, cellulose, and surprisingly pectin and their resultant effects on recalcitrance [9–25].
- BESC led large-scale campaigns to understand natural variation in both switchgrass and *Populus*. This included both high-throughput (HTP) recalcitrance phenotyping and sequencing along with other omics for these natural variants (as well as the generated transgenics). These resulted in advances in genome-wide association studies (GWAS) [26–28].
- BESC conducted greenhouse and field trials for a limited number of *Populus* and switchgrass lines with reduced recalcitrance arising from both directed transgenics and natural variants. We utilized a collective “TOP Line” experimental design protocol [29] with multiple phenotypic characterization assays developed by the Enabling Technology groups. These included sugar release, sugar and lignin composition, ethanol production, crystallinity, etc. A key discovery was the ability to achieve both lower recalcitrance and higher biomass simultaneously in certain lines [25, 30–32].
- One goal of BESC was to understand the molecular basis of recalcitrance. The reduced recalcitrance feedstock biomass generated in BESC was analyzed by a series of chemical, biochemical, molecular, and systems biology approaches. The outcome was the identification of multiple wall polymers whose modified abundance or structure could be engineered to reduce feedstock recalcitrance [8]. The results begin to provide mechanistic understanding of the molecular bases of recalcitrance.
- From this research, we can see a path for improving feedstocks by cisgenic manipulations, by selecting the best natural variants, or by genetically assisted breeding [33].

### Biomass conversion

One-step Consolidated Bioprocessing (CBP) without added enzymes [34] was the central focus of BESC’s work in the conversion area, which featured both fundamental and applied components. BESC initially focused on two approaches (a) improving product formation in thermophilic cellulolytic bacteria (primarily *Clostridium thermocellum* and *Caldicellulosiruptor bescii*), and (b) conferring to yeasts the ability to ferment cellulose by virtue of heterologous expression of glycosyl hydrolases. We came to regard the former approach as more promising and by the end of BESC were focused exclusively on this path. Key conversion advances led by BESC included:

- Large differences were found among the biocatalysts in the most comprehensive comparative evaluation to date of biomass deconstruction under controlled conditions. Among the biocatalysts tested, thermophilic anaerobes and specifically *C. thermocellum* achieved the highest carbohydrate solubilization yields which were several-fold higher yields than industry-standard fungal cellulase [35–37].
- We developed and improved the genetic tools for thermophiles, most notably *C. thermocellum* and *Caldicellulosiruptor* spp., and use of these tools to initiate metabolic engineering of these non-model microbes [38–41].
- Substantial advances were made in understanding and manipulating the metabolism of target CBP microbes. Zhou et al. [42], described non-standard glycolysis in *C. thermocellum*. *Thermoanaerobacter saccharolyticum* was improved to produce economically recoverable ethanol concentrations at near-theoretical yield in the hemicellulose-fermenting [43]. Iso-butanol was produced by adding key pathway enzymes in modified *C. thermocellum* at unprecedented yields and titers [44]. Ethanol titer and yield were increased in *C. thermocellum* by elimination of side-products [45–47].
- BESC identified the specific deconstruction enzymes which target the major biopolymers of lignocellulosic biomass. Work on enzyme fundamentals emphasized multifunctional cellulases based on the enzymes found in *Caldicellulosiruptor* species and *C. thermocellum* [48]. CelA, a multifunctional glycosyl hydrolase from *C. bescii*, was shown to be a particularly powerful hydrolytic enzyme despite being inhibited by the presence of lignin [49, 50].
- BESC demonstrated that *C. thermocellum* is capable of active fermentation in the presence of mechanical milling—an approach referred to as co-treatment [35, 51, 52]. With co-treatment, *C. thermocellum* was able to achieve greater than 85% carbohydrate solubilization for *Populus* and switchgrass in the absence of added enzymes and thermochemical pretreatment implying that *C. thermocellum* can attack all the major chemical linkages in representative woody and herbaceous lignocellulose crops given sufficient physical access.

### Enabling technology

Enabling technologies were organized to develop and apply cutting-edge analytical methodologies to characterize biomass as well as its conversion. There was also significant omics and computational biology of the modified plants and microbes to help improve metabolic models. The resulting data were used to create new insights into how biomass structure and chemistry affect recalcitrance during CBP or pretreatment. These efforts included analyses of partially digested solid residues from CBP.

- The development of high-throughput methods for rapid analysis of pretreatment and enzymatic hydrolysis allowed for rapid identification of low recalcitrant plant lines from thousands of natural and transgenic variants. These low recalcitrant plant lines then could be characterized using multiple analytical and omic approaches which rapidly advanced BESC’s deeper understanding of the recalcitrance phenotype [53–55].
- Increased understanding of recalcitrance was supported by developing techniques such as glycome profiling [56], and improving the use of nuclear magnetic resonance spectroscopy (NMR) for biomass [12, 57–60].
- BESC also supported the development of new ways to image the chemical components comprising the cell wall. Raman spectroscopy was used to image hemicellulose for the first time [61]. Modified AFM techniques were used to chemically image the cell wall at the submicron level [62]. Quantitative fluorescence CLSM and surface spectroscopy by ToF–SIMS showed , following microbial digestion, the decrease in surface cellulose while surface lignin increased. This indicates that biomass recalcitrance may be controlled by surface characteristics [63].
- Co-solvent enhanced lignocellulosic fractionation was developed as a new pretreatment that removes significant amounts of lignin and increases enzymatic digestibility of biomass [64].
- The center was able to provide integrated omics data for key processes. Integrated omics of microbial growth on complex lignocellulosic biomass over time provided a detailed view of the molecular machinery (metabolites and enzymes) and revealed temporal adaptation to a complex, lignocellulose substrate [65]. Profiling genotype-specific proteomes derived from RNA sequencing data better defined the link between genotypes and phenotypes in *Populus* [66].
- Lignin has been shown to play a key role in biomass recalcitrance [67–69]. The potential removal or recovery of lignin would allow its valorization [70] whether into fiber [71] or into value-added intermediates [72].

## Structures and management

As a thematically rather than institutionally defined center, BESC recognized early on the need to develop a shared organizing vision, a sense of priority, and strong mechanisms for shared samples and data as well as strong management. This structure allowed us to recruit many of the nation’s experts in recalcitrance and to draw on the intellectual cultures and strengths of different institutions. BESC successfully implemented a flexible management approach, modeled after successful biotech startup companies that have relied on academic research to strengthen their science base and industrial partnerships to translate discoveries into commercial products.

Over the decade, BESC established a distinctive, high functioning collaborative team with participants from 22 institutions and a broad range of disciplines. As needs and research progressed, six partners left the center and five new partners joined. BESC included researchers from universities, national laboratories, and private companies. These partners included major efforts at ORNL, University of Georgia, Athens, and the National Renewable Energy Laboratory. Specialized expertise was provided by Dartmouth College, Georgia Tech, University of Tennessee, Knoxville, Cornell University, West Virginia University, University of California-Riverside, University of California-Los Angeles, North Carolina State University, University of North Texas along with The Samuel Roberts Noble Foundation (Noble). Earlier partners included Brookhaven National Laboratory, University of Minnesota, Washington State University, and Virginia Polytechnic Institute. Our industrial partners included DuPont, Mascoma Corporation, Diversa Corporation, ArborGen, Inc., Ceres, Inc., and GreenWood Resources, Inc.

BESC brought together individuals, institutions, and disciplines to focus on understanding and ameliorating biomass recalcitrance. As a result of discussions at our retreats and other fora, ideas emerged that would not have happened without a center so designed. As a result of common management, resources, and non-disclosure agreements, barriers to collaboration were substantially lowered as compared to individual investigators acting on their own. Students and postdoctoral staff were among the greatest beneficiaries. Upon hiring BESC-supported students, companies observed that they had extraordinary experience functioning as part of interdisciplinary teams. As an indication of the extent of collaboration, about half of our publications in year 9 had co-authors from more than one BESC institution.

Top-down structures and bottom-up networking were useful and complementary in fostering integration. Weekly calls were held by the science and operational management team. Twice-monthly calls were held with a larger group (roughly 15) consisting of the science management and activity leads, with topics alternating between science and management calls. BESC refreshed its management, all but two of the original eight management team members being replaced by year 10; this included hiring a new Director. Keeping leadership fresh was also accomplished as early and mid-career staff—some of them graduate students at the start of BESC—were promoted and given added responsibility such that by the end of BESC they comprised over half of BESC activity and project leads. Task- and organism-specific points of contact were designated to facilitate interactions among teams and individuals.

## Technology transfer and outreach

Technology transfer, managed by a Commercialization Council chaired by ORNL, and consisting of the COO and technology transfer leads from each partner institution operated as a community of best practice to strategically and effectively engage with industrial partners. A “storefront” on the BESC website provided a centralized online portal for industry to view available technologies for licensing and partnering. The IP management plan was built on a common Inter-Institutional Agreement template that allows a designated lead institution to offer jointly owned IP from multiple BESC members [73]. Another assessment of the value of the advances is shown by technoeconomic evaluation of several advanced disruptive improvements; CBP with co-treatment was projected to have a potential eight-fold improved return-on-investment [51].

Tech transfer metrics at the time of writing featured more than 190 invention disclosures resulting in 60 patent applications and 21 executed licenses. For example, in 2016, two companies licensed a gene discovered using GWAS in *Populus trichocarpa* [74]. GreenWood Resources plans to utilize the gene to select low lignin poplar variants for further breeding resulting in lower-cost improvements in either conversion processes or pulping. Forage Genetics Intl. will commercialize this genetic mechanism to reduce lignin and increase desirable flavonoids. This will increase digestibility and the nutritional value of animal feedstocks such as alfalfa, corn, and sorghum.

The ability to freely share materials and protect potential IP that belong to the BESC partners is an essential function for expeditious collaboration within the Center. A laboratory information management system (LIMS) served as the main mechanism for documenting the transfer of materials among BESC partners as allowed under the innovative BESC Master Material Transfer Agreement. The LIMS also represented the primary system for tracking large experimental campaigns, protocols, data, and metadata and for data quality assurance. LIMS is a relatively mature system developed during BESC using a commercial LIMS software package, Nautilus (http://www.thermo.com), which was specifically designed to manage flexible laboratory processes. This system has an Oracle relational database engine as its back end and generated numerous customized workflows and web interfaces to view results from laboratory processes and experimental campaigns, which has successfully been used to track more than 100,000 samples during the BESC project.

The nationwide BESC Outreach program targeted science enrichment and educational standards in 4th–6th grades. In collaboration with the Creative Discovery Museum in Chattanooga, Tennessee, we developed a hub-and-spoke model using hubs at 18 national museums and science centers in 14 states (Utah, Idaho, Montana, New Mexico, Kansas, Oregon, Washington, Georgia, Tennessee, Alabama, Texas, Michigan, Illinois, Florida, and Oklahoma) [75]. The “Farming for Fuels” Program is available on our websites. Over 225,000 students, parents, and teachers have participated in hands-on activities. These are not hits on a website, they are person-to-person contacts and educational activities. The enhanced Biofuels website (http://www.learnbiofuels.org) with information and downloadable biofuels-related lesson plans has received more than 45,000 page views. A Biofuels/Alternative Energy iPad software app “Road Trip Challenge” is available through the iTunes App Store with eight “trips” between hub museums. Importantly, the program is moving closer to becoming self-sustaining. Of the over 50,000 students, teachers, and parents reached during 2016, 81% were served with no direct program-support cost to BESC.

## Summary

BESC has redefined biomass recalcitrance from an observable phenotype to a better understood and manipulatable fundamental and operational property. These manipulations are the result of deeper biological understanding and can be combined with other advanced biotechnology improvements in biomass conversion [76, 77] to improve bioenergy processes and markets.
